# Ammonium Removal in Aquaponics Indicates Participation of Comammox *Nitrospira*

**DOI:** 10.1007/s00284-021-02358-3

**Published:** 2021-02-05

**Authors:** Julia Heise, Hubert Müller, Alexander J. Probst, Rainer U. Meckenstock

**Affiliations:** Environmental Microbiology and Biotechnology, University of Duisburg-Essen, Universitätsstr. 5, 45141 Essen, Germany

## Abstract

Aquaponic systems are sustainable solutions for food production combining fish growth (aquaculture) and production of vegetables (hydroponic) in one recirculating system. In aquaponics, nitrogen-enriched wastewater from fish in the aquaculture serves as fertilizer for the plants in the hydroponics, while the nitrogen-depleted and detoxified water flows back to the aquaculture. To investigate bacterial nitrogen-cycling in such an aquaponic system, measurements of nitrogen species were coupled with time-resolved 16S rRNA gene profiling and the functional capacity of organisms was studied using metagenomics. The aquaponic system was consistently removing ammonia and nitrite below 23 µM and 19 µM, and nitrate to steady-state concentrations of about 0.5 mM. 16S rRNA gene amplicon sequencing of sediments exposed in the pump sump revealed that typical signatures of canonical ammonia-oxidising microorganisms were below detection limit. However, one of the most abundant operational taxonomic units (OTU) was classified as a member of the genus *Nitrospira* with a relative abundance of 3.8%. For this genus, also genome scaffolds were recovered encoding the only ammonia monooxygenase genes identified in the metagenome. This study indicates that even in highly efficient aquaponic systems, comammox *Nitrospira* were found to participate in ammonium removal at low steady-state ammonia concentrations.

**Electronic supplementary material:**

The online version contains supplementary material available at (10.1007/s00284-021-02358-3).

## Introduction

Over the last 50 years, aquaponic systems have become a promising biotechnology for sustainable food production. In aquaponics, aquaculture (fish cultivation) and hydroponics (vegetables production) are integrated into one water-circulating system. One of the main issues in aquaculture is the accumulation of ammonium due to decomposed fish food and fish excrements. Depending on the fish species and the exposure time [1], ammonia is chronically toxic to fish in concentrations higher than 1.9 mg/L total ammonia nitrogen at pH 7 and 20 °C. For the common carp (Cyprinus carpio), it was shown that even an ammonia concentration of 1.3 mg/L can be mortal to small individuals of about 10 g [2]. Therefore, the water of aquaculture systems must be replaced regularly or requires cleaning. Aquaponic systems solve the issue of regeneration by pumping water from the fish tank into the grow beds of the hydroponic part, where it serves as fertilizer for vegetables, fruits, or herbs. Plants can use both ammonium and nitrate as nitrogen sources [3]. However, for lettuce, pak choi, tomato, and chive, it was shown that nitrogen is mainly assimilated in the form of nitrate [4].

The essential reaction in the aquaponic system is the nitrification during which toxic ammonium is oxidised to less harmful nitrate, which can be carried out by different microorganisms [5, 6]. It was shown that low pH can shift microbial communities in an aquaponic system [7]. Since the complete oxidation of ammonia to nitrate is an essential step for the effective operation in an aquaponic system, the nitrogen turnover in such a system was elucidated in this study.

Until 2015, nitrification was assumed to be exclusively performed in a two-step process carried out by two phylogenetically distinct bacterial lineages. In the first step, ammonium is oxidised to nitrite via hydroxylamine by e.g. *Nitrosomonas*. In the second step, nitrite is oxidised to nitrate by e.g. *Nitrobacter* or *Nitrospira*. This process was observed in marine [8, 9] and freshwater [10–13] aquaponic or aquaculture systems by enrichment techniques, fluorescence in situ hybridization, or sequence analyses.

In 2015, Daims et al. [14] and van Kessel et al. [15] discovered that nitrification can also be carried out by one single organism affiliated to the genus *Nitrospira*. By showing that this bacterium was capable of oxidising ammonium fully to nitrate, a process called ‘comammox’ (complete ammonia oxidation), the authors overturned the 100-yr-old lasting paradigm that nitrification can only be performed by two distinct groups of organisms. Since their discovery, comammox *Nitrospira* have been frequently detected in aquifers [16], drinking water systems [17], wastewater treatment plants [18], as well as in recirculating aquaculture systems [19]. Furthermore, 16S rRNA gene analysis of community compositions in different compartments of an aquaponic system showed that nitrification took place on a biofilter located behind the fish tank retaining large particle matter [20]. Since *Nitrospira* was among the most abundant species and other nitrifying bacteria seemed not to be present in the biofilm community of the aquaponic system, the authors assumed that the nitrification process was carried out by *Nitrospira* alone.

So far, all known comammox *Nitrospira* belong to the sublineage II of *Nitrospira* [14, 15, 17], which comprises comammox *Nitrospira* species and nitrite-oxidising (canonical) *Nitrospira* species [21, 22]. Additionally, comammox and canonical *Nitrospira* form mixed phylogenetic clades within this sublineage [14, 15, 17], suggesting that they cannot be distinguished based on 16S rRNA sequences alone.

Here, we aim at elucidating the type of microbial nitrogen metabolism in an aquaponic system and how the microbial communities develop over time. A combination of 16S rRNA gene sequencing and metagenomic analysis is employed for distinguishing different types of nitrification and to follow the respective functional clades.

## Material and Methods

### The Aquaponic System

**Fig. 1:**
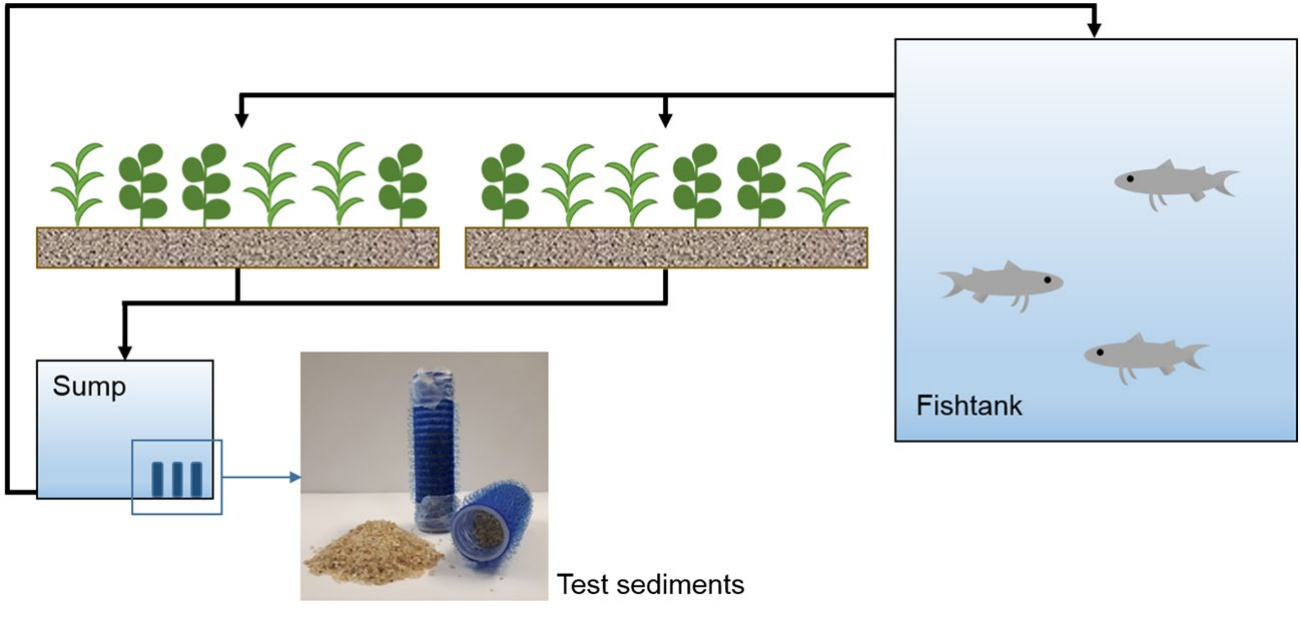
Schematic view of the backyard aquaponic system (not to scale). The test sediments used for analysing the microbial community were exposed in small cylinders in the sump (hair curlers)

The aquaponic system investigated in this study was a private backyard system. The fish tank was built from a regular 1200 L plastic intermediate bulk container (IBC) and filled with 1000 L tap water (Fig. 1). The hydroponic part consisted of two grow beds, each 100 × 120 cm in size and filled with gravel up to 30 cm in height. The overflow water from the fish tank flew into the grow beds by gravity and was treated by a biofilter made of gauze with 1 mm mesh size to remove larger particles. The particle filter was cleaned daily. The water of the grow beds was periodically released into a sump by a hydraulic siphon system. From there, it was continuously pumped back into the fish tank with an adjusted flow rate of 800 L/h. The aquaponic system was installed in the open and environmental conditions such as exposure to sunlight and temperature were not controlled. Water losses due to evapotranspiration were compensated by refilling with tap water.

In the beginning of the operation, five common carps (Cyprinus carpio) and five wels catfish (Silurus glanis) were grown corresponding to a fish density of 10 fish/cm^3^. After a fish disease killing all the fish, the water in the aquaponic system was completely exchanged and all system parts were cleaned. Only ten carps were grown subsequently resulting in individuals of 400 g weight each at the end of the experiment. The fish were fed with dried honey bee larvae. Four Lettuce, three tomatoes, two cucumber, four chard, and three strawberries were grown in the grow beds from May to October.

### Preparation of Test Sediments and Sampling

Quartz sand (1 mm grain size) was autoclaved for 20 min at 120 °C and introduced into the pump sump as substratum for microbial growth. Regular plastic hair curlers (6.5 cm in length) with porous walls (1 × 0.5 mm pore size) were filled with approximately 10 g of the sand and closed with parafilm at both ends. The test sediments were incubated in the aquaponic sump. In total, 12 curlers were taken for amplicon sequencing over a period of 508 days ending in October. For metagenome sequencing, DNA was extracted from one test sediment taken at the last day of incubation. Water was sampled on all sampling days to check the system performance and analyses of nitrogen species concentrations (see below). The pH was determined onsite using test stripes (pH 4.5–10, Carl Roth GmbH, Karlsruhe, Germany) to guarantee a steady pH over time.

### Chemical Analysis

Water samples were regularly taken from the aquaponic sump and concentrations of nitrate, nitrite, and ammonium were analysed in triplicates with ion chromatography. The samples were prepared by 1:2 dilution with 0.01 mM potassium buffer for the analysis of anions and with 20 mM methyl sulfonic acid (70% v/v) (Merck KGaA, Darmstadt, Germany) for the analysis of cations. All samples were centrifuged for 15 min at 16,000×*g* to remove solids. 200 µL of the supernatants were diluted 1:5 with ultrapure water (18.2 µS, Merck Millipore System, Merck KGaA, Darmstadt, Germany). The samples were stored at − 20 °C until analysis.

Anion and cation concentrations were measured with an ion chromatograph (Thermo Fisher Scientific, Dreieich, Germany) equipped with a Dionex™ IonPac™ AS23-4 µm column and an AERS 500 suppressor (2 mm) for the measurement of anions using 0.8 mM NaHCO_3_/4.5 mM Na_2_CO_3_ (Thermo Fisher Scientific, Dreieich, Germany) as eluent at a flow rate of 0.25 mL/min at 7 mA. Ammonium was measured with a Dionex™ IonPac™ CS12 A column and a CERS 500 suppressor (2 mm) using 20 mM methyl sulfonic acid (70% v/v) (Merck KGaA, Darmstadt, Germany) as eluent at a flow rate of 0.25 mL/min at 15 mA. The detection limit was calculated based on the calibration method according to DIN EN 32645. Non-equidistant calibration points (10, 20, 50, 100, 200 µM) were used.

### DNA Extraction

DNA was extracted from the sand of each curler using the FastDNA™ SPIN Kit for Soil (MP Biomedicals, Heidelberg, Germany). 460–480 mg of sand (wet weight) were taken and treated as described in the manufacturers’ instructions. For the cell lysis, the bead-beating system Precellys24 tissue homogenizer [Bertin Instruments, Montigny-le-Bretonneux, France] was used for two times at 65,000 rpm for 30 s each turn. The DNA samples were stored at − 20 °C until further usage.

### Preparation of 16S rRNA Gene Amplicon Library

The preparation of the amplicon library was adapted from the Illumina 16S sequencing library preparation guide (part no. 15044223 Rev. B). The primers Pro341F/Pro805R [23] targeting the V3-V4 region of 16S rRNA genes of bacteria and archaea were applied to get 250 bp reads lengths. They were combined with the Illumina overhang adapters (Eurofins Genomics, Ebersberg, Germany).

For the first stage PCR, 2 µL of extracted DNA was mixed with 1X KAPA HiFi Hot Start Ready Mix (Roche, Basel, Switzerland), 0.25 μM of each the forward and the reverse primers including the Illumina overhang adapters, and nuclease-free water (Qiagen, Hilden, Germany) to a final reaction volume of 25 µL. Duplicates were prepared for each sample and pooled after the PCR. The PCR amplification was carried out with an initial denaturation step at 94 °C for 5 min followed by 30 cycles of denaturation at 94 °C for 30 s, annealing at 55 °C for 30 s, and extension at 70 °C for 1 min, and a final extension at 70 °C for 5 min.

The PCR amplicons were purified using MagSi-NGS^PREP^ Plus magnetic beads (Steinbrenner, Wiesenbach, Germany) by thoroughly mixing 32 µL of magnetic beads with 40 µL of samples and following the PCR clean-up instructions given in the Illumina 16S metagenomic sequencing library preparation guide with the exception that the beads were resuspended in 42.5 µL of elution buffer EB (Qiagen, Hilden, Germany). 40 µL of the supernatants were then taken for further analyses.

The index PCR was performed using the Nextera XT DNA Library Preparation Kit v2 Set D (FC-131-2004) from Illumina (Munich, Germany). The PCR and the second PCR clean-up were performed as described in the Illumina 16S metagenomic sequencing library preparation guide.

DNA concentrations were measured with a Qubit fluorometer using the Qubit™ dsDNA HS Assay Kit (ThermoFisher Scientific, Dreieich, Germany). The samples were normalised to a concentration of 2 ng/µL and 5 µL of all samples were combined into one ready-to-load sample containing 12 libraries, which was analysed by GATC Biotech AG (Konstanz, Germany) on an Illumina Miseq platform. The 16S rRNA gene sequence reads are deposited in the NCBI’s nr database as SUB5504898 in the bioproject PRJNA534201.

### Analysing 16S rRNA Gene Sequencing Data

The 16S rRNA sequences were analysed as paired-end run using the mothur-based MetaAmp Version 2.0 software [24]. The settings were similarity cutoff of 0.97, minimum overlap of 35 bp, no mismatches in the overlap region, no differences in primer sequences, max. one expected error, and a trim amplicon length of 350 bp. The alignment of reads was conducted using the SILVA 128 database.

Statistical analyses were carried out using the R code described in [25]. For multivariate statistics, data were rarefied (to the lowest number of sequences in the samples, which was 173,469 reads per sample) and a Bray–Curtis distance was calculated. In order to account for rarefication biases, this procedure was repeated 100 times and the distance across these iterations was averaged. Alpha diversity was determined using the Shannon index. Principle coordinate analysis (PCoA) was used to display the beta diversity of the samples. Relationships between OTUs and environmental factors (time, pH, nitrate, ammonia, sulphate, chloride, sodium, magnesium, and calcium) were calculated using PERMANOVA (Adonis testing).

Identification of OTUs that significantly correlated with time in relative abundance were selected by applying a Pearson correlation. Only OTUs with a *P*-value < 0.001 are reported in the manuscript.

### Metagenomic Sequencing and Analysis

To study functionalities, 390 ng of DNA were extracted from the sand sample on the last sampling day for the whole metagenome sequencing. Library preparation and Illumina HiSeq sequencing of paired-end 150-bps reads were done by GATC Biotech AG (Konstanz, Germany). Raw reads obtained from GATC Biotech were trimmed and quality filtered using bbduk (http://jgi.doe.gov/data-and-tools/bbtools/) and SICKLE version 1.21 (https://github.com/najoshi/sickle). The processed reads were assembled and scaffolded using metaSPADES version 3.10.1 [26]. For scaffolds longer than 1 kb genes were predicted using prodigal [27] and diamond blastp [28] was used to annotate the genes against the Unifref100 database [29], which contained taxonomy information from UniProt and the NCBI taxonomy database.

Databases of 100 amino acid sequences each for ammonia monooxygenase subunit A (*amoA*), subunit B (*amoB*), and subunit C (*amoC*) and the hydroxylamine reductase were created from highly identical sequences derived from NCBI’s nr database. The sequences were aligned using MUSCLE [30] and maximum likelihood phylogenetic trees were constructed based on the JTT matrix-based model [31] using the MEGA7 software [32] by applying default settings.

## Results

### Nitrogen Species in the Aquaponic System

**Fig. 2:**
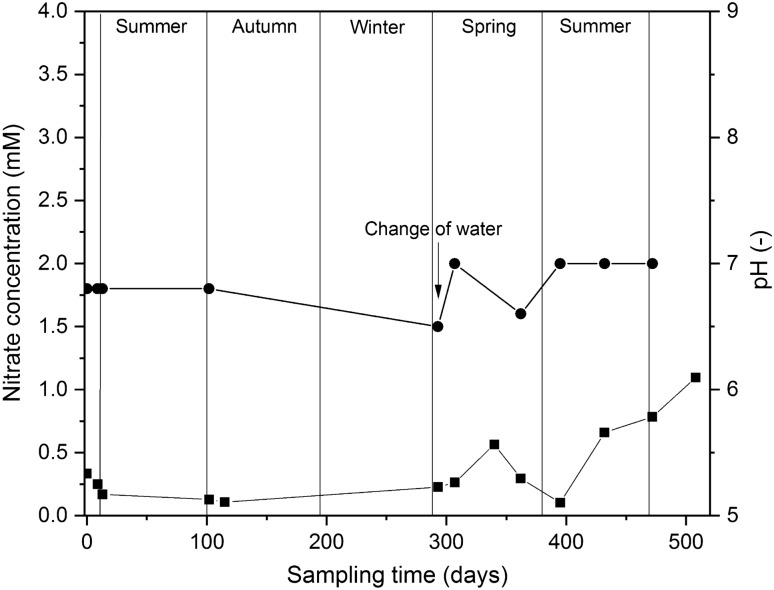
Concentrations of nitrate (squares) and pH (circles) in the backyard aquaponic system from May 2016 to October 2017. Concentrations of nitrite and ammonium were below the detection limits of 23 µM and 19 µM, respectively, throughout the monitoring time

The aquaponic system efficiently removed nitrogen from the water. Only nitrate was measurable in low concentrations up to 1.1 mM in the sump of the aquaponic system at the end of the system operation (Fig. 2). Ammonium and nitrite concentrations were always below 23 µM and 19 µM, respectively, indicating that both ammonium and nitrate were taken up by the plants and residual ammonium was completely oxidised by nitrifying microorganisms. In April 2017, the concentration of nitrate raised to 0.6 mM due to the starting metabolism of the fish resulting in a higher nitrogen load of the water. When seedlings were planted in May, nitrate and potentially ammonium were increasingly taken up by the plants leading to a decrease of nitrate concentrations to 0.1 mM in June 2017. The nitrate concentrations increased again from June onwards to 1.1 mM at the end of the operation in October 2017. The absence of ammonium in all samples indicated that a nitrifying microbial community was established in the aquaponic system. The pH stayed constant between 6.8 and 7 and was not adjusted.

### Microbial Diversity

Sequencing of 16S rRNA genes revealed a highly diverse community structure in the aquaponic system. A total of 40 bacterial phyla were identified, out of which 15 made up 95.4% of the total microbial community (Supplementary material Figure S1). The most dominant phyla in the aquaponic system were Proteobacteria, Bacteroidetes, Verrucomicrobia, Acidobacteria, Actinobacteria, and Nitrospira. The three most abundant OTUs classified on family level at the end of the operation of the aquaponic system (day 508) were *Verrucomicrobiaceae* (4.3%), *Nitrospiraceae* (3.8%), and *Comamonadaceae* (2.8%), which belong to the phylum Proteobacteria.

**Fig. 3:**
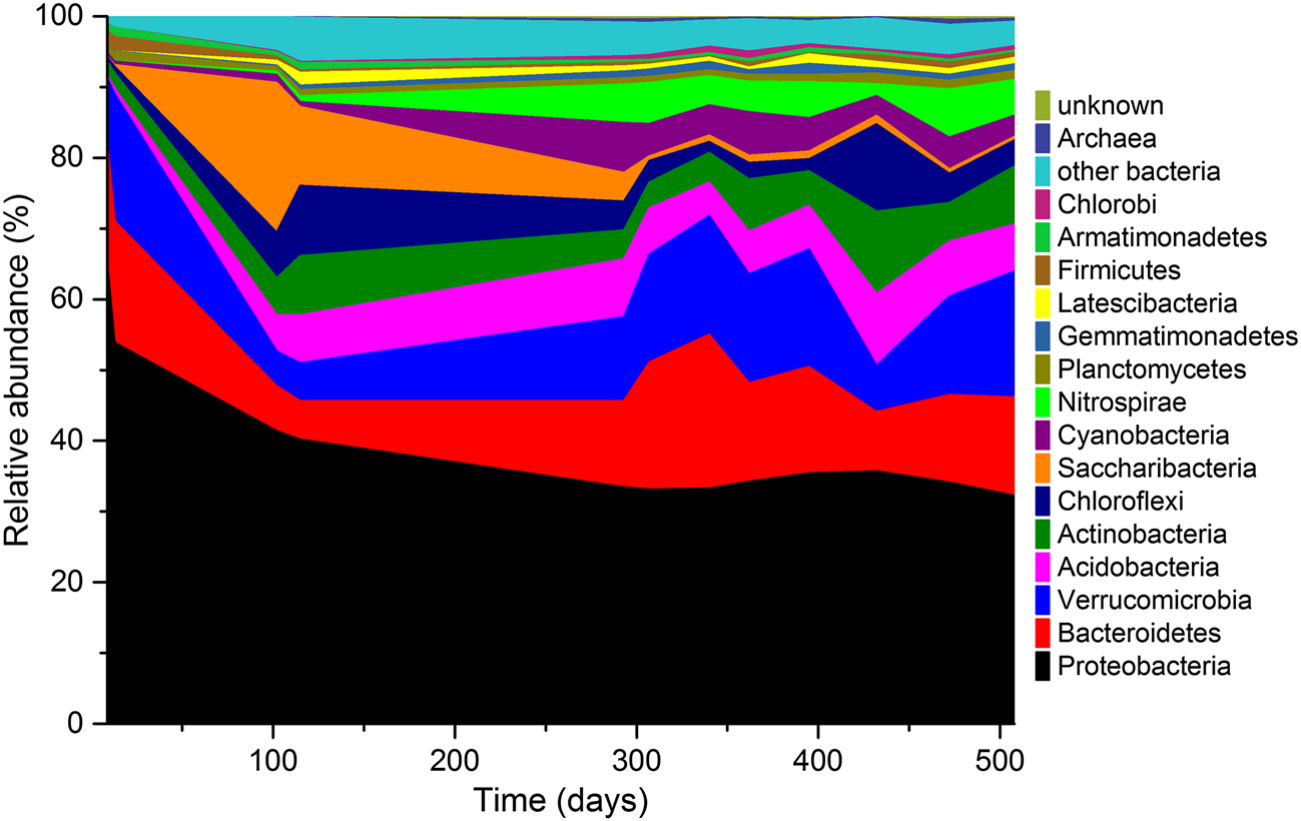
Relative abundances of the 100 most abundant taxonomic genera in the aquaponic system based on 16S rRNA gene sequencing. Taxonomy was sorted based on the total reads of the sample “day 508”. Please consider the repetition of colours. A water exchange was performed on day 293. Other bacteria summarise all bacteria that could be classified but do not belong to the 15 most abundant bacterial taxa or Archaea. Unknown depicts all putative OTUs that could not be assigned to known taxa

In the highly diverse samples, the 100 most abundant genera make up almost 50% of the total community at the end of the operation of the aquaponic system (Fig. 3). After a water change on day 293, unclassified *Verrucomicrobia*, unclassified *Chloroplasts*, as well as *Nitrospira*, *Aquabacterium*, and *Arenimonas* were among the most dominant genera based on OTUs. The dominant OTU classified as *Nitrospira* was found in all samples after the water change. The classical genus of ammonia oxidisers, *Nitrosomonas*, was only found in very low relative abundance (0.03%) suggesting that the detected *Nitrospira* were fully oxidising ammonium to nitrate similar to the previously found *Candidatus* Nitrospira inopinata [14, 15].

**Fig. 4:**
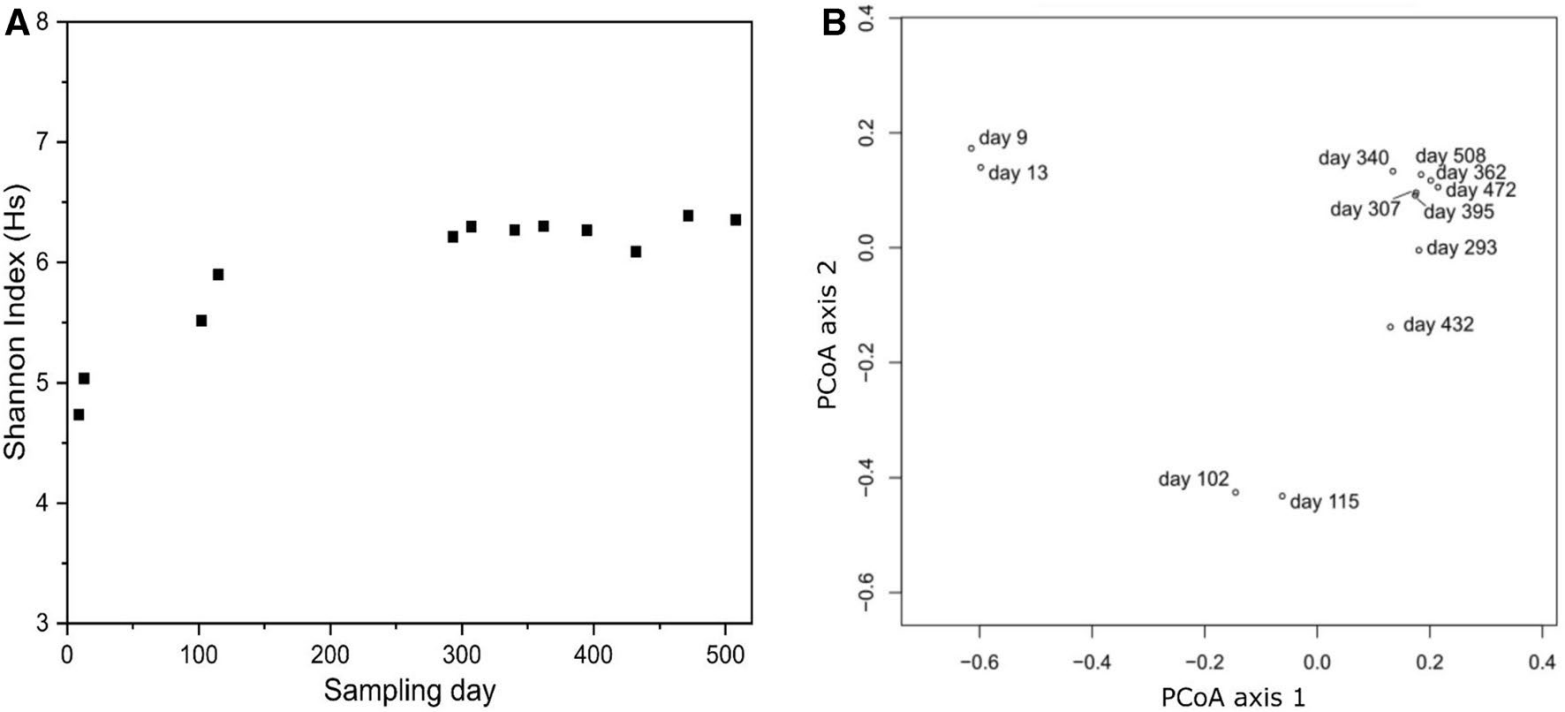
Shannon indices (**a**) as measure for microbial alpha diversity calculated from OTU abundances at different time points. Principal coordinate analysis diagram (**b**) showing the beta diversity of OTUs in the microbial community at different sampling days. Principal coordinate analysis axis 1 and axis 2 explained 44.2% and 22.5% of the total variance in the community, respectively

The microbial diversity in the aquaponic system was described using the Shannon index H_S_ (Fig. 4a). During the first year of operation, the diversity was increasing over time from 4.74 (day 9) to 6.30 (day 307). During the second year, the Shannon index stagnated between 6.27 and 6.39 indicating a stabilisation of the microbial diversity on a very high diversity level. A total water exchange was performed on day 293.

Principal coordinate analysis of the relative abundances of OTUs showed a distinct shift in the microbial community composition over time (Fig. 4b), which can be clustered in early, mid, and late time samples. Samples taken in the beginning of the operation in spring 2016 (day 9 and day 13) differed significantly from the samples taken in the midpoint of the operation at the end of summer (day 102 and day 115). After the change of water in spring 2017 (day 293), the late time cluster microbial community composition deviated again showing a precedent shift in the winter period. In the last half of the year of operation, the community composition seemed to become more stable and shifted only slightly with seasons.

As indicated in the principal coordinate analysis, time had a substantial impact on the microbial community composition (PERMANOVA *P*-value 0.001). Interestingly, no other measured parameter showed a significant association with the observed microbiome structure. OTUs classified as members of the genera *Nitrospira*, *Acidobacteria*, *Sphingobacteria,* and *Cytophagia* were less abundant or not even detectable in the beginning and increased continuously during the operation time of the aquaponic system (Fig. 3). Other genera, mainly belonging to the *Alphaproteobacteria*, decreased in abundances over time. However, even after one and a half years of operation, a steady-state community had not been reached according to the PERMANOVA test.

### Metagenome Analyses

**Fig. 5:**
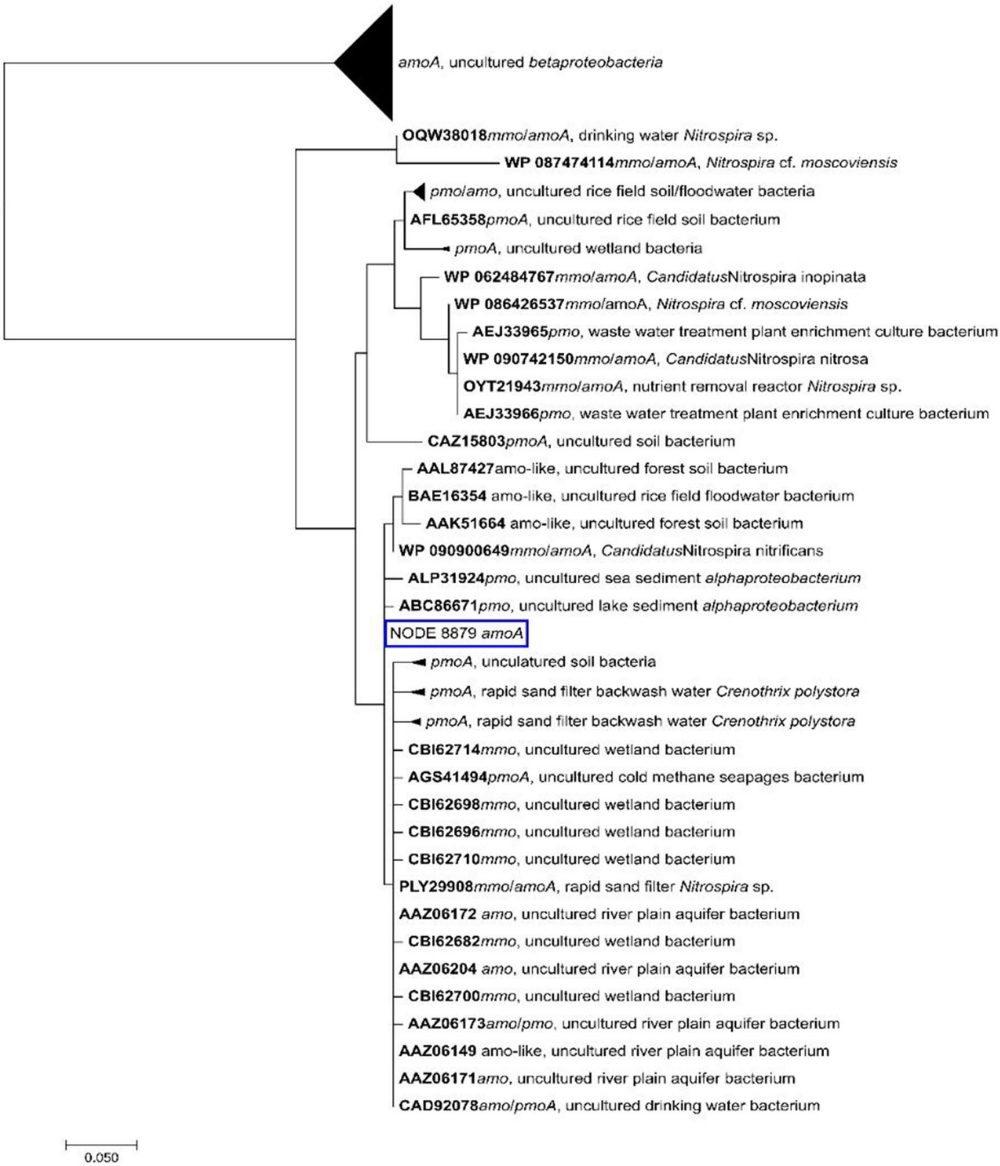
Phylogenetic tree of *amoA* gene sequences using the maximum likelihood method based on the JTT matrix-based model [31]. *Amo* ammonia monooxygenase. *Mmo* methane monooxygenase. *Pmo* particulate methane monooxygenase. Scale bar indicates estimated number of substitutions per site

Metagenome sequencing was performed from the samples taken at the last sampling day (day 508) to investigate the metabolism of the key players in ammonia oxidation. The assembled metagenome showed that the ammonia monooxygenases genes *amoA*, *amoB*, and *amoC* were only found on scaffolds classified as *Nitrospira*. The ammonia monooxygenase subunits *amoA* (Fig. 5), *amoB*, and *amoC* (Supplementary material Fig. S3 and S4) showed highly identical amino acid sequences to those obtained from reference sequences of comammox *Nitrospira nitrificans* (Table 1). The sequences of amino acids showed highest identities (98% for *amo* subunits and 96% for hydroxylamine reductase) with comammox *Nitrospira* species found on a rapid sand filter and a household sand filter [33, 34]. Two further scaffolds contained *amoA* and *amoC* genes, respectively (Supplementary material Table S1).

**Table 1:** Comparison of genes involved in complete ammonia oxidation obtained in this study and from known strains

	*Nitrospira nitrificans*		*Nitrospira nitrosa*		*Nitrospira inopinata*	
	Identity	Score	Identity	Score	Identity	Score
*amoA*	WP_090900649.1		WP_090742150.1		WP_062484767.1	
	98%	499	91%	437	90%	443
*amoB*	WP_090900646.1		WP_090742146.1		WP_062484768.1	
	93%	782	82%	723	82%	734
*amoC*	WP_090895910.1		WP_090744476.1		WP_062484140.1	
	98%	351	96%	342	95%	340
*hcp*	WP_090894739.1		WP_090900629.1		WP_062481664.1	
	70%	564	69%	561	70%	566

Identity and total score were retrieved from a blast search against the NCBI’s nr database.

Moreover, hydroxylamine reductases were only found in *Nitrospira* (Supplementary material Figure S5). Two additional genes were identified for a putative hydroxylamine reductase (Supplementary material Table S1). Consequently, *Nitrospira* was the only significantly abundant genus capable of ammonia oxidation in the backyard aquaponic system.

## Discussion

### Performance of the Aquaponic System

The free ammonia concentration is one of the most important parameters in aquaculture since it is toxic to fish species in relatively low concentrations (1.3 mg/L for common carps) [2]. In an aquaponic system, free ammonia nitrogen is released into the tank by the fish and taken up by the plants in the grow beds either as ammonium or after oxidation to nitrate [3]. The nitrifying microbial community in the sediments converts the residual ammonium that is not taken up by plants to nitrate, which is less harmful to fish species than ammonia [5, 6]. Although nitrification and degradation of organic carbon lead to acidification, the gravel of the grow beds obviously buffered the system well enough to keep the pH constant. The crucial concentration of ammonium is at the end of the hydroponic part at the influent to the aquaculture fish tank. In our aquaponic system, a nitrifying community was efficiently established reducing the ammonium concentration effectively below 23 µM. However, the nitrate concentration is rising during summer and the begin of autumn exceeding the concentration of 23 µM. It seems that more ammonium was produced due to the fish growth and less nitrogen was assimilated into the plants, which were at their end phase of growth and food production. The surplus of ammonium was then converted most likely by comammox *Nitrospira*. These results can be underlined by the fact that the abundances of OTUs assigned to the genera *Nitrospira* correlated positively with time meaning they became more abundant during the end of the operation of the aquaponic system.

Nitrification in aquaculture biofilters is most efficient at slightly alkaline pH of 7.5 to 9.0 [35, 36]. However, hydroponic plants grow best at slightly acidic conditions such as pH 5.5 for romaine lettuce crops [37] and 5.5 to 6.0 for greenhouse cucumber [38]. Compromising the efficiencies of nitrification, fish cultivation and plant growth, neutral pH 7 is commonly accepted as the optimal pH for aquaponic systems [39]. Our open aquaponic system was continuously running at pH 6.8–7.0. The steady pH may arise from the high adjusted flow rate of 800 L/h so that ammonium could not accumulate in the fish tank. An adjustment was not needed providing an efficiently self-regulated system with optimal nitrogen removal.

### Microbial Community Composition

Since the focus was on microbial nitrification processes, the test sediments installed in the pump sump were located between the grow beds and the fish tank. Until now, microbial diversity in aquaculture and aquaponic systems was mainly studied on biofilters [11, 12, 19, 20, 40]. In aquaponic systems, however, the biofilter function is replaced by the grow beds.

In our test sediments, the most abundant OTUs were member of *Proteobacteria*, *Bacteroidetes*, *Verrucomicrobia*, *Acidobacteria* and *Actinobacteria*. The same phyla were also among the most dominant ones of an aquaponics biofilter community [20]. In contrast, Bartelme et al. [19] identified *Actinobacteria*, *Gammaproteobacteria*, *Planctomycetes*, and *Sphingobacteria* as the most dominant phyla in freshwater recirculating aquaculture systems (RAS). Sugita et al. [12] found *Alphaproteobacteria*, *Betaproteobacteria*, *Nitrospira*, *Actinobacteria*, *Bacilli*, *Gammaproteobacteria*, *Planctomycetacia*, and *Sphingobacteria* as predominant phyla in similar systems. However, nitrification also takes place in the grow beds, which was not assessed here.

Surprisingly, the microbial community compositions in the aquaponic system changed continuously over 508 days. Since the parameters determined for the aquaponic system were relatively constant, the change of microbial community compositions only correlated with the factor time. However, this study focusses on the nitrification in an aquaponic system. The role of other bacteria that correlate with the factor time remains unclear.

There was no correlation detectable between the nitrifying microorganisms and nitrate concentrations. Since the ammonium concentration was always very small, the removal of the nitrogen compounds by the plants was in equilibrium with the ammonia production by the fish and the microbial nitrification keeping all nitrogen species at low steady-state concentrations.

### Two-Step Nitrification or Comammox?

The microbial community composition on the sediments revealed *Nitrospira* as the only organisms potentially involved in nitrification. The classically known members of the genus *Nitrospira* perform the second part of the two-step nitrification, the oxidation of nitrite to nitrate. For a complete nitrification process, a second organism such as *Nitrosomonas* would be essential to oxidise ammonia to nitrite. However, ammonia-oxidising bacteria like *Nitrosomonas* were at negligible abundance in our aquaponic sediments. *Nitrospira* not only belonged to the most abundant organisms in the aquaponic system but was also most likely performing the complete ammonia oxidation to nitrate. Similar results were reported by Schmautz et al. [20], who found *Nitrospira* to be one of the most abundant species in the aquaponic system. Since other ammonia-oxidising bacteria were only found at very low abundance, the authors assumed that the detected *Nitrospira* were comammox organisms [20].

Ammonia is first oxidised to hydroxylamine by ammonia monooxygenase, which consists of at least three subunits (*amoA*, *amoB*, and *amoC*). Hydroxylamine is then oxidised to nitrite catalysed by the hydroxylamine reductase. These genes can, therefore, be taken as indicators for ammonia oxidation. Detection of both genes for ammonia and nitrite oxidation in one genome are strongly indicative of comammox [14]. In our aquaponic system, the scaffolds of genes for the ammonia-oxidising enzymes were only found on scaffolds classified as *Nitrospira*. *Nitrosomonas* und *Nitrobacter*, which have been regarded as the main nitrifying bacteria so far, do probably not play a pivotal role in nitrification in our freshwater aquaponic systems. Hence, the interpretation of the community analysis was strongly supported by the metagenomic analysis and the detection of genes coding for enzymes involved in nitrification.

Comammox *Nitrospira* were predicted to survive in environments with low ammonium concentrations [41]. This assumption was recently underlined, when comammox *Nitrospira* were detected in groundwater [16], drinking water systems [17, 33] as well as recirculating aquaculture systems with low ammonium loading [19]. Nitrification allows for only little energy conservation requiring a high substrate turnover for microbial growth [41]. Hence, the cells have to produce lots of enzymes dedicated to nitrification and fit those into the limited cell volume. At high ammonia concentrations and high growth rates, it might become beneficial that the whole nitrification process is shared as partial processes between the ammonium oxidizer and the nitrate oxidizer because they can produce higher rates if they fill the limited cell volume with the enzymes for only one partial process. The overall rate is then higher. At very low ammonia concentrations, however, comammox bacteria obtain an advantage because they grow at low rates, do not need that many enzymes, and can thus fit both processes for complete oxidation into one cell [41].

Due to the high similarities of the genes involved in complete ammonia oxidation between known comammox *Nitrospira* and those obtained in this study, their high abundance, and the non-detection of classical ammonia oxidizers, we conclude that ammonia was mainly oxidised by comammox *Nitrospira* in the aquaponic system.

## Electronic supplementary material

Below is the link to the electronic supplementary material.

Electronic supplementary material 1 (DOCX 756 kb)
