# Supplementary material for: Ammonium Removal in Aquaponics Indicates Participation of Comammox Nitrospira
Source: Curr Microbiol. 2021 Feb 5;78(3):894–903. doi: 10.1007/s00284-021-02358-3 (PMC7952344; doi:10.1007/s00284-021-02358-3)
Supplement: Supplementary file 1 — Electronic supplementary material 1 (DOCX 756 kb) [file 284_2021_2358_MOESM1_ESM.docx]

**Supplementary material**


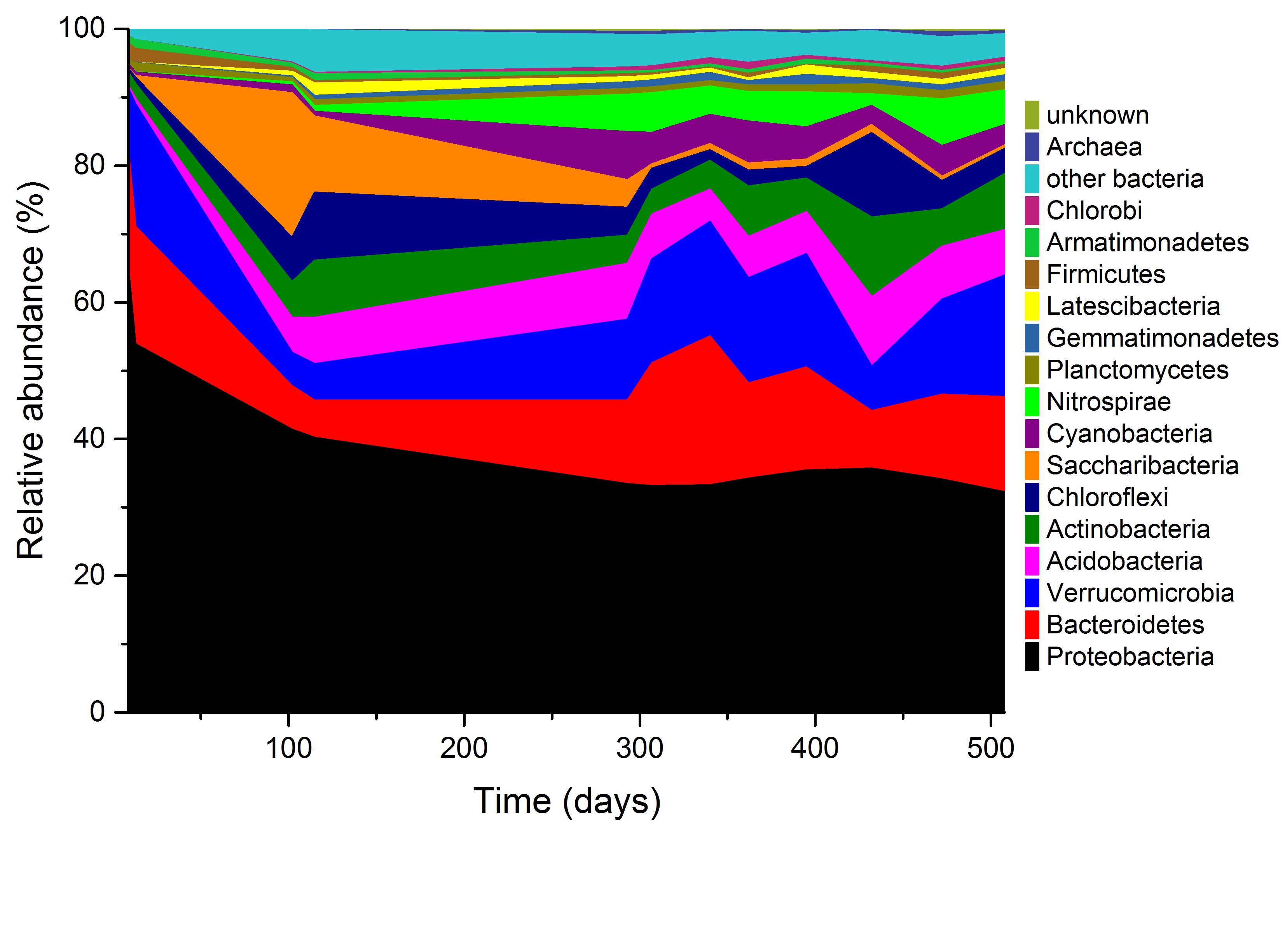


Figure S1: Relative abundances of taxonomic phyla in the aquaponic system based on 16S rRNA gene sequencing. Phyla with abundances less than 0.5% of total reads were grouped as “other bacteria”. Taxonomy was sorted based on the total reads. Sequences assigned to unknown bacteria are called “other bacteria”. If sequences could not be assigned remain “unknown”.


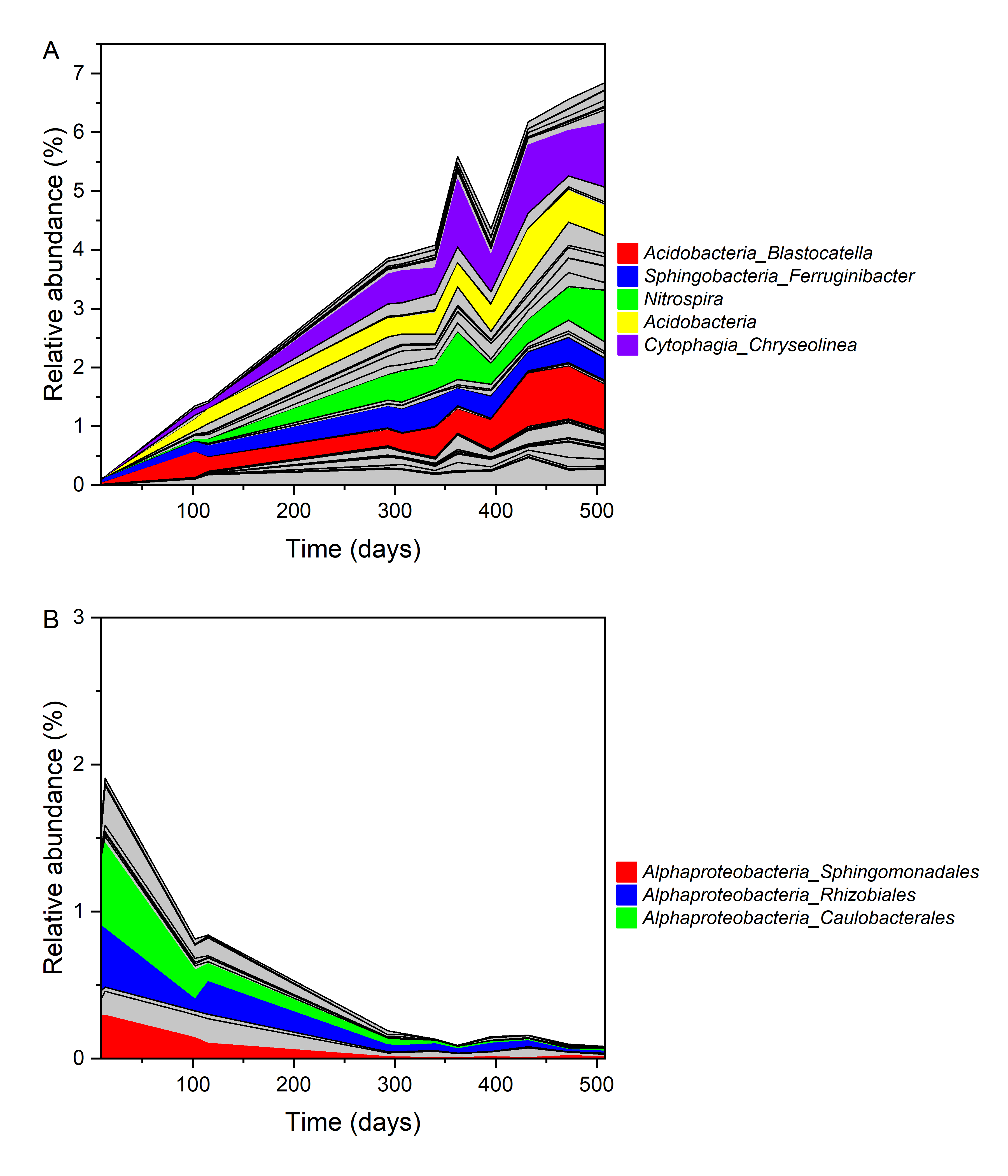


Fig. S2. Correlation of main OTUs with the factor time. Relative abundances of 50 OTUs are shown in each panel that changed either most positively (A) or most negatively (B) changed with time. Note the differences in scales. The coloured graphs depict the main OTUs which are strongly correlating with time. OTUs that correlate less with time are shown in grey.


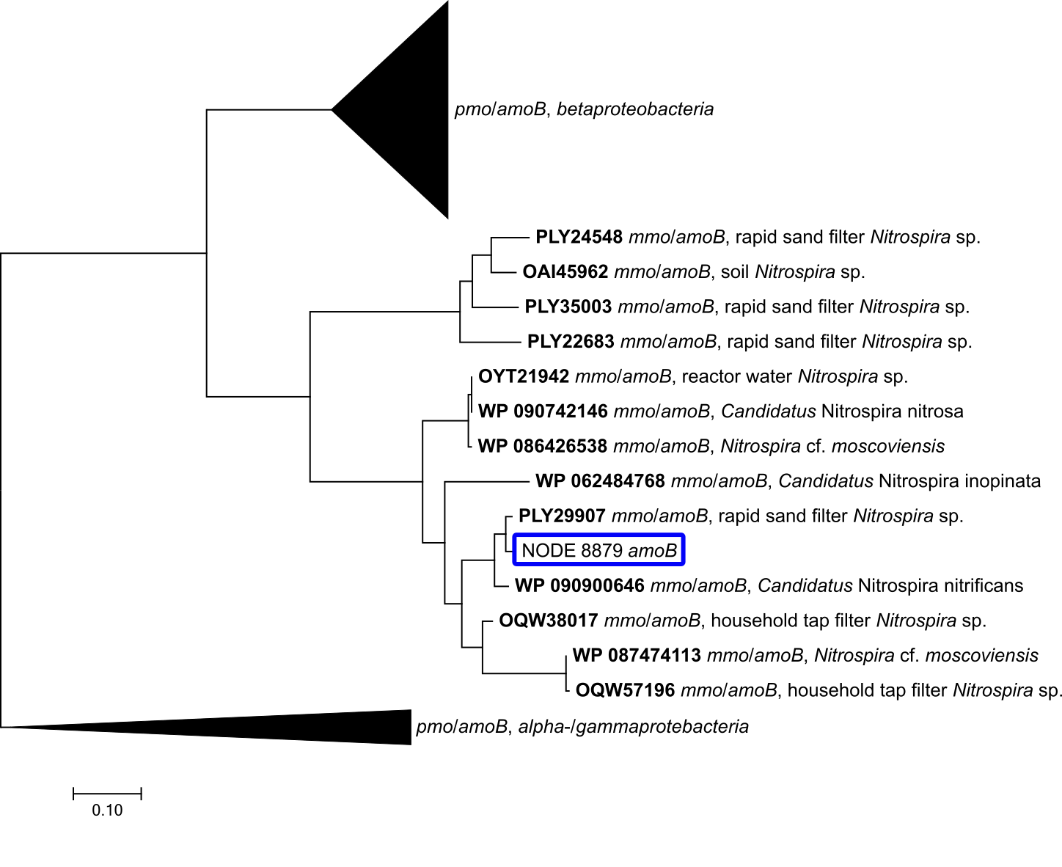


Fig. S3. Phylogenetic tree of *amoB* gene sequences using the maximum likelihood method based on the JTT matrix-based model (Jones *et al.*, 1992). *Amo*: ammonia monooxygenase. *Mmo*: methane monooxygenase. Scale bar indicates estimated number of substitutions per site.

*
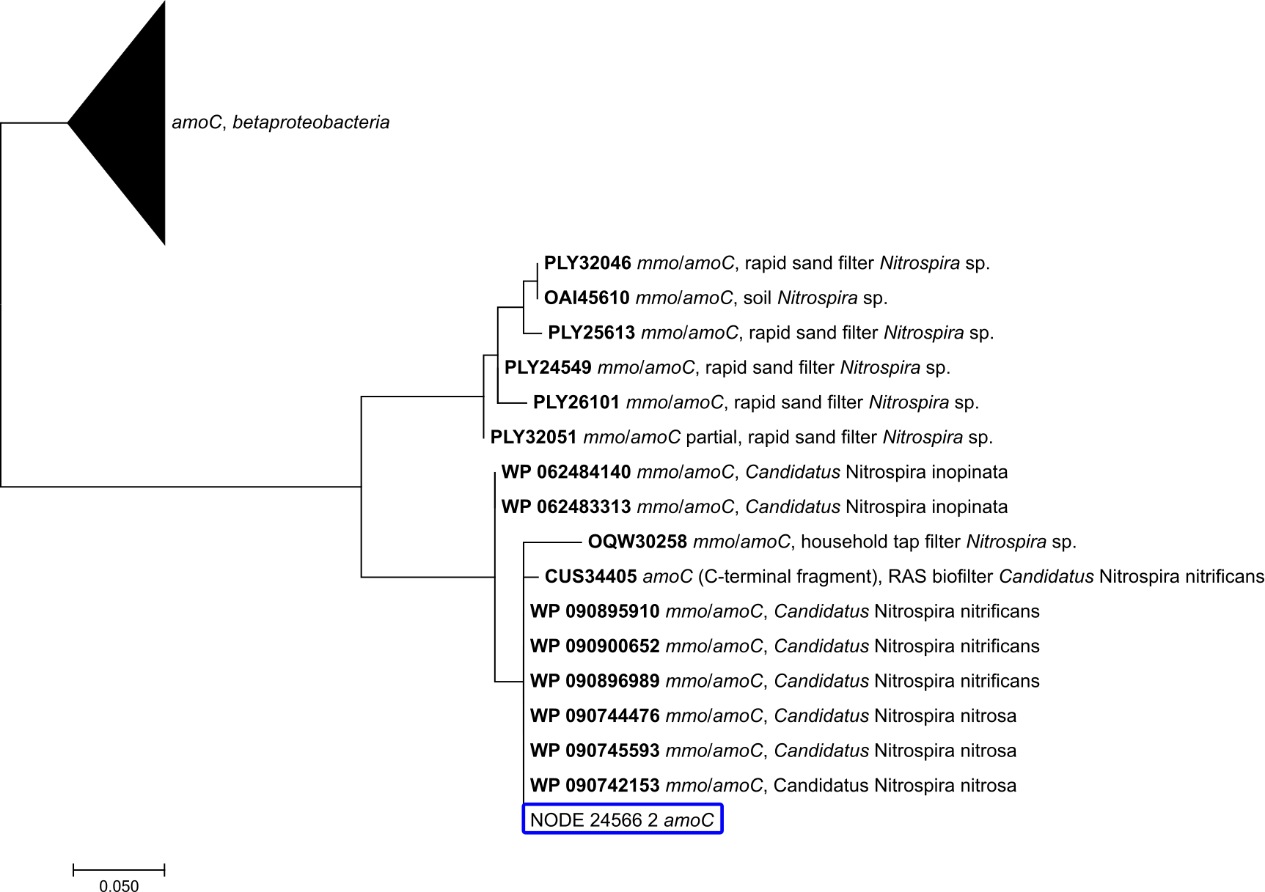
*

Fig. S. Phylogenetic tree of *amoC* gene sequences using the maximum likelihood method based on the JTT matrix-based model (Jones *et al.*, 1992). *Amo*: ammonia monooxygenase. *Mmo*: methane monooxygenase. Scale bar indicates estimated number of substitutions per site.


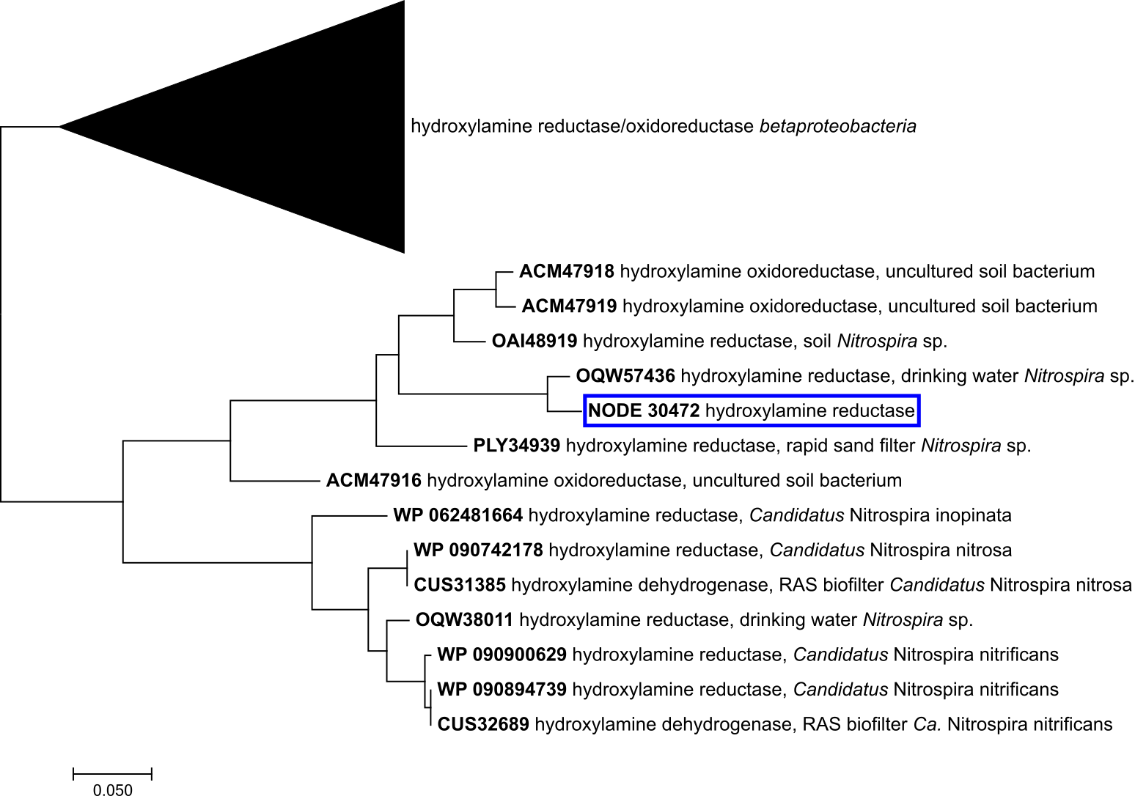


Fig. S5. Phylogenetic tree of hydroxylamine reductase gene sequences using the maximum likelihood method based on the JTT matrix-based model (Jones *et al.*, 1992). RAS: recirculating aquaculture system. Scale bar indicates estimated number of substitutions per site.

Table S1. Genes annotated as ammonia monooxygenase and hydroxylamine reductase.

Table S2. Predicted amino acid sequences of genes annotated as ammonia monooxygenase and hydroxylamine reductase.
